# Optimization of Method for Pesticide Detection in Honey by Using Liquid and Gas Chromatography Coupled with Mass Spectrometric Detection

**DOI:** 10.3390/foods9101368

**Published:** 2020-09-26

**Authors:** Mariana O. Almeida, Silvia Catarina S. Oloris, Vanessa Heloisa F. Faria, Márcia Cassimira M. Ribeiro, Daniel M. Cantini, Benito Soto-Blanco

**Affiliations:** 1 Instituto Otávio Magalhães, Fundação Ezequiel Dias (Funed), Rua Conde Pereira Carneiro 80, Belo Horizonte 30510-010, MG, Brazil; mariana.almeida@funed.mg.gov.br (M.O.A.); silvia.oloris@funed.mg.gov.br (S.C.S.O.); vanessa.faria@funed.mg.gov.br (V.H.F.F.); marcia.marcos@funed.mg.gov.br (M.C.M.R.); daniel.menegati11@gmail.com (D.M.C.); 2 Departamento de Clínica e Cirurgia Veterinárias, Escola de Veterinária, Universidade Federal de Minas Gerais (UFMG), Avenida Antônio Carlos 6627, Belo Horizonte 30123-970, MG, Brazil

**Keywords:** residues in food, pesticides, LC-MS/MS, GC-MS/MS, QuEChERS, honey

## Abstract

This study aimed to optimize and validate a multi-residue method for identifying and quantifying pesticides in honey by using both gas and liquid chromatographic separation followed by mass spectrometric detection. The proposed method was validated to detect 168 compounds, 127 of them by LC-MS/MS (liquid chromatography tandem mass spectrometric detection) and 41 by GC-MS/MS (gas chromatography tandem mass spectrometric detection). The limit of detection (LOD) and limit of quantification (LOQ) values for the analytes determined by LC-MS/MS were 0.0001–0.0004 mg/kg and 0.0002–0.0008 mg/kg, respectively. For GC-MS/MS analyses, the LOD and LOQ values were 0.001–0.004 mg/kg and 0.002–0.008 mg/kg. In total, 33 samples of commercial honey produced by apiaries in six Brazilian states were analyzed with the validated method. Residual amounts of 15 analytes were detected in 31 samples (93.9%). The method described in the present study was able to detect an extensive and broad range of pesticides with very high sensitivity.

## 1. Introduction

Honey is consumed by humans worldwide because of its characteristic sweet flavor and as a medicinal food. It is produced by honeybees, mainly from nectar collected from flowers. However, honey may be contaminated with pesticides used on crops foraged by bees. Contamination may occur through direct contact of the bee body to the pesticide or by bee consumption of the contaminated nectar, pollen, and guttation fluid (an exudate eliminated through the tips or edges of leaves of some plants) [1,2,3]. Furthermore, some pesticides are used to treat beehives against diseases [4].

The consumption of residual pesticides in contaminated foods has been linked to several toxic effects in humans, such as carcinogenesis, immunological disorders, and neurological disturbances [5]. Maximum residue levels (MRLs) have been established for pesticides in honey to ensure consumers’ safety [6,7,8,9]. It is mandatory to avoid the commercialization of honey containing residual pesticides at levels above the MRLs. To determine residual pesticide levels, precise and sensitive analytical methods must be able to detect an extensive and broad range of compounds.

Several analytical methods have been developed for detecting single compounds to a few dozen pesticides in honey. In these methods, detection and quantification are performed using techniques such as liquid chromatography (LC) with diode array [10], ultraviolet [11,12], fluorescence [13], and electrochemical [11] detectors, gas chromatography (GC) with electron capture [14], flame ionization [15], nitrogen–phosphorus [16], flame photometric [17], thermionic-specific [18], and atomic emission [19] detectors, and excitation–emission matrix fluorescence data [20].

The performance of chromatographic analysis depends on adequate sample extraction and cleanup procedures. Matrix compounds are concentrated at the extraction procedure, whereas interfering substances are removed by the cleanup procedure [21]. An innovative technique developed for sample extraction and cleanup procedures is the QuECHERS (Quick, Easy, Cheap, Effective, Rugged, and Safe) method [22]. Compared to earlier procedures, this method reduces the volume of solvents, and offers practical performance. Modifications of the QuECHERS method have been used for the detection of pesticides in different matrices such as meat [23], fish [24], milk [25], and honey [3,26,27,28,29,30,31].

Simultaneous detection of the residual levels of several pesticides in honey is mandatory in several countries to inspect this food before commercialization. Multi-residue analysis of at least one hundred pesticides in honey has been achieved using LC and GC coupled to mass spectrometric (MS) or tandem mass spectrometric (MS/MS) detection [1,3,26,27,28,29,30,31,32].

This study aimed to develop and validate a multi-residue method for identifying and quantifying pesticides in honey by using both gas and liquid chromatographic separation followed by mass spectrometric detection.

## 2. Materials and Methods

### 2.1. Chemicals and Reagents

Acetonitrile, ethyl acetate (both high performance liquid chromatography [HPLC] grade), and formic acid (for analysis) were supplied by Merck (Darmstadt, Germany). Methanol (HPLC grade) was obtained from Honeywell (Charlotte, NC, USA). Ammonium formate (>99%) was purchased from Vetec (Rio de Janeiro, Brazil). A DisQuE^TM^ CEN sample preparation kit in pouch format (each pouch containing 4.0 g of anhydrous magnesium sulfate, 1.0 g of sodium chloride, 1.0 g of trisodium citrate dihydrate, and 0.5 g of disodium hydrogen citrate sesquihydrate; all > 99%) was supplied by Waters (Milford, CT, USA). An ExtraBond^®^ QuEChERS Dispersive kit EN (each tube containing 900 mg of anhydrous magnesium sulfate and 150 mg of primary and secondary amine (PSA); both > 99%) was obtained from Scharlab (Barcelona, Spain). D-Sorbitol (≥98%) and gluconolactone (>99%) were purchased from Sigma–Aldrich (Darmstadt, Germany). Ultrapure water was generated with a Millipore Milli-Q system (Milford, CT, USA). All reference standards were of high purity grade (>98.0%) and were obtained from Dr. Ehrenstorfer (Augsburg, Germany) or AccuStandard (New Haven, CT, USA). Individual stock solutions were prepared at an approximate concentration of 1000 ng/µL in acetonitrile or acetone and stored in a freezer at −20 °C. Working solutions were prepared through appropriate dilutions of the stock solutions.

### 2.2. Samples

Blank samples of honey were obtained from apiaries managed under an organic system, and repeated analyses confirmed the absence of residual pesticides. These blank samples were fortified with target analytes for the validation of the analytical method. Furthermore, 33 samples of commercial honey produced by apiaries in six Brazilian states (Distrito Federal, Goias, Minas Gerais, Rio Grande do Norte, Rio Grande do Sul, and São Paulo) were analyzed using the validated method.

### 2.3. Sample Preparation

The modified QuEChERS method for extraction and cleanup was optimized from previously described procedures [22,28,32,33]. Each honey sample (5.0 g) was placed into a 50 mL polypropylene tube and spiked with appropriate amounts of pesticides in working solutions. Next, 10.0 mL of ultrapure water was added, and the mixture was agitated at 1750 rpm for 2 min. Exactly 10.0 mL of a solution of acetonitrile and ethyl acetate (70:30, *v/v*) was added, and each tube was agitated again at 1750 rpm for 2 min. Then, 4.0 g of anhydrous magnesium sulfate, 1.0 g of sodium chloride, 1 g of trisodium citrate dehydrate, and 0.5 g of disodium hydrogen citrate sesquihydrate were added, and the tubes were agitated at 1750 rpm for another 2 min and centrifuged at 4000 rpm for 5 min. The whole organic layer was transferred to a 15 mL polypropylene tube, and the mixture was kept at −40 °C for at least 2 h. The supernatant (6.0 mL) was mixed with 900 mg of anhydrous magnesium sulfate and 150 mg of PSA, and the mixture was agitated at 1750 rpm for 1 min and centrifuged at 3600 rpm for 5 min. The extract (4.0 mL) was transferred to two 13 × 100 mm glass tubes, with 2.0 mL in each tube. The solution was dried in an evaporator with a water bath maintained at 45 °C and nitrogen pressure of 15 psi.

The procedural internal standard (P-IS) [34] for the LC analysis was Propoxur, and the P-IS for GC analysis was 4,4´-dichlorodiphenyldichloroethylene (4,4´-DDE). After weighing the honey sample, 10 μL of the P-IS solution containing 4.0 ng/μL of Propoxur and 4.0 ng/μL of DDE 4,4 was added. Propoxur and 4,4´-DDE were then validated following the validation method described in Section 2.3.

For LC analysis, the dried residue was reconstituted with 200 µL of methanol:water (1:1), with both solvents containing 5 mM ammonium formate and 0.01% formic acid. After 30 min, the tube was vortexed for 1 min, and the solution was transferred to a vial containing a conical insert of 250 μL.

For GC analysis, the dried residue was reconstituted with 200 µL of acetonitrile:ethyl acetate (7:3) and 6 µL of analyte protectant solution, composed of 10 mg/mL gluconolactone and 5 mg/mL D-sorbitol in acetonitrile:water (7:3). The tube was then immediately vortexed for 0.5 min, and the solution was transferred to a vial containing a conical insert of 250 μL.

### 2.4. Liquid Chromatography

LC-MS/MS (liquid chromatography tandem mass spectrometric detection) analysis was performed using an Agilent 6495 Triple Quadrupole LC/MS system. Chromatographic separations were carried out on a Zorbax SB-C18 Rapid Resolution HT column (4.6 × 150 mm, 1.8 µm) at a 40 °C column temperature. The mobile phases were water containing 5 mM ammonium formate and 0.01% formic acid (phase A) and methanol containing 5 mM ammonium formate and 0.01% formic acid (phase B), with gradient elution at a flow rate of 0.6 mL/min. The gradient elution program was as follows: 0 min, 90% B; 2.0 min, 50% B; 20 min, 100% B. The total chromatographic run time was 25 min. The injection volume was 5 µL.

For mass spectrometric analysis, an electrospray ionization (ESI) source was used in both negative (ESI-) and positive (ESI+) modes. Source parameters were set as follows: gas temperature 120 °C, gas flow 15 L/min, nebulizer 45 psi, sheath gas flow 12 L/min, sheath gas temperature 300 °C, capillary voltage 3500 V (+ and −), nozzle voltage 300 V (+)/500 V (−), iFunnel RF high pressure 150 V (+)/90 V (−), and iFunnel RF low pressure 60 V (+ and −). The retention times, delta retention times, polarities, ion transitions, and collision energies are presented in Table 1. Two transitions were chosen for almost all pesticides, but an extra confirmatory transition was included for four pesticides to avoid false-positives at trace pesticide levels. The analysis was run according to all requirements for identifying analytes by MS/MS established by European Union SANTE/12682/2019 [34].

### 2.5. Gas Chromatography

GC-MS/MS (gas chromatography tandem mass spectrometric detection) analysis was performed using an Agilent 7000C Triple Quadrupole GC/MS system with a multimode inlet. The temperature of the injector was maintained at 150 °C (0.1 min), ramped up to 300 °C at 600 °C/min (20 min hold), and then ramped down to 200 °C at 20 °C/min until the end of the analysis. The injection volume was 2 μL. The pulsed splitless injection was at 50 psi for 0.5 min with a split flow of 50 mL/min for 0.6 min. The gas saver was set to 20 L/min and started after 5 min. The carrier gas was helium, and the inlet pressure was 5.59 psi (constant pressure mode) during the run and 2.0 psi during the backflush. From the inlet, two Agilent HP-5ms Ultra Inert (5%-phenyl)-methylpolysiloxane columns (0.25 mm, 0.25 µm) were coupled to each other through a purged ultimate union for post-run backflushing; the first column was 30 m, and the second column was 2 m. The total chromatographic run time was 29.5 min, and backflushing started after 25.5 min with 8.92 psi. The column oven temperature was maintained at 60 °C for 1.0 min, ramped up to 180 °C at 30 °C/min, and then ramped up to 300 °C at 5 °C/min.

For the mass spectrometric analysis, an electron ionization source was used with an ionization voltage of 70 eV, ion source temperature of 290 °C, and interface temperature of 280 °C. The retention times, delta retention times, polarities, ion transitions, and collision energies are presented in Table 2. Two transitions were chosen for almost all pesticides, but an extra confirmatory transition was included for seven pesticides to avoid false-positives at trace pesticide levels. The analysis was run according to all requirements for identifying analytes by MS/MS established by European Union SANTE/12682/2019 [34].

### 2.6. Method Validation

Validation was performed following the European Union SANTE/12682/2019 [34] and Codex Alimentarius CXG90-2017 [35] guidelines. The following analytical performance parameters were assessed: linearity, selectivity, trueness, precision (repeatability and within-lab reproducibility), limit of detection (LOD), and limit of quantification (LOQ). A total of 209 different analytes were tested, 159 of them by LC-MS/MS and 50 by GC-MS/MS.

Matrix-matched calibration (MMC) was used to minimize the matrix effect. For the preparation of analytical MMC curves, blank honey extracts were spiked with appropriate amounts of standard solutions at the six final concentrations. Three independent solutions were prepared for each level of the curve (*n* = 18), and the samples were injected randomly. The difference between the calculated concentration and the theoretical concentration must be less than or equal to 20% for the curve’s best fit. The selectivity was determined by identifying the pesticide in the presence of the matrix and other analytes. If interfering peaks were detected at the same retention time as some pesticides, the interfering agents’ areas had to be less than or equal to 30% of the analyte LOQs.

The trueness and precision (repeatability and within-lab reproducibility) were determined from the recovery assay results of blank samples spiked with all of the analytes at two distinct levels (LOQ and 10× LOQ) for GC-MS/MS and three distinct levels (LOQ, 2× LOQ, and 10× LOQ) for LC-MS/MS. Repeatability was evaluated using data from replicate samples (*n* = 6) analyzed on the same day for each level. The within-lab reproducibility was evaluated using replicate data (*n* = 12) from two different days and two analysts for each level. Repeatability and within-lab reproducibility are expressed by the relative standard deviation (RSD in %), whereas average recovery values express trueness. The expanded measurement uncertainty (U) was estimated by the top-down approach. All results are reported in Table 3 and Table 4. Average recovery ranging from 70% to 120% was considered adequate. Precision deviations of up to 20% were considered acceptable [34].

The LOQ was determined as the lowest concentration level of the calibration curve with acceptable accuracy. The LOD corresponded to 50% of the estimated value for the quantification limit, provided that the recoveries presented an area greater than or equal to 50% of the point in the matrix solution injected and that the signal/noise ratio was higher than or equal to 3.

## 3. Results and Discussion

### 3.1. Extraction Method

The extraction procedure is a crucial step for detecting pesticides, and it can be challenging for a complicated matrix such as honey. Extraction procedures that have been developed for honey samples include solvent extraction, supercritical fluid extraction, solid-phase extraction, matrix solid-phase dispersion, solid-phase microextraction, stir bar sorptive extraction [36], purge and trap, dispersive liquid–liquid microextraction, microextraction by packed sorbent, single-drop microextraction, magnetic solid-phase extraction [37], and solvent floatation [38]. In the present method, the QuEChERS method was optimized for the extraction and cleanup of honey samples from the original method [22] with modifications for honey [28,33] and bee pollen samples [32]. The original QuEChERS method consists of an extraction step with acetonitrile and separation using extraction salts, followed by a cleanup step with purification salts [22].

Different extraction and cleanup conditions were evaluated for this method. Honey samples were diluted in water prior to extraction. Acetonitrile:ethyl acetate (70:30, *v/v*) solution provided better extraction efficiency, similar to Souza Tette et al. [28]. On the other hand, Mitchell et al. [33] used acetonitrile:water (50:50, *v/v*) solution without the sample’s previous dilution. In the present study, the extracted solution was subjected to freeze-out before the dispersive solid phase extraction (d-SPE) cleanup, following the method developed for bee pollen by Vázquez et al. [32]. The extraction recoveries for most pesticides were improved by keeping the extract in the freezer at −40 °C for at least 2 h (Supplementary Materials Table S1). Furthermore, extracted solutions that were subjected to freeze-out were visually more translucid than solutions that were not subjected to freeze-out.

The cleanup procedure of the present study was performed with magnesium sulfate and PSA. The same purification salts were also used by Mitchell et al. [33], but at different amounts (150 mg magnesium sulfate and 100 mg PSA); in contrast, Souza Tette et al. [28] also included Florisil (50 mg) to magnesium sulfate (150 mg) and PSA (50 mg). The extract was concentrated ten times after cleanup to achieve lower LOD and LOQ values, similarly to an earlier study [33]. The effectiveness of the modifications to the QuEChERS method in the present study was confirmed by the wide range of pesticides successfully detected and the high sensitivity evidenced by the low LOD and LOQ values.

### 3.2. Validation Assay

The proposed method was validated to detect 168 compounds, 127 of them by LC-MS/MS and 41 by GC-MS/MS. The matrix effect was minimized by using MMC. The method’s selectivity was determined by identifying the pesticide in the presence of the matrix and other analytes. All validated compounds showed average recoveries ranging from 70% to 120%. The mean repeatability relative standard deviation (RSD) for all samples in the LC-MS/MS method was 7.75%, ranging from 2% to 20%, and in the GC-MS/MS method the RSD was 7.24%, ranging from 3% to 15%. The expanded measurement uncertainty (U) for all samples in the LC-MS/MS method was 11.4%, ranging from 3% to 20%, and in the GC-MS/MS method was 13.1%, ranging from 4% to 20%. Average recoveries ranging from 70% to 120% and precision RSD of up to 20% were considered adequate [34]. The estimation of the uncertainty of an analytical method can be performed in different ways, including empirical, practical, or top-down approaches [39]. In the present study, the uncertainty was estimated using the top-down approach. In this way, the experimental design to estimate the RSD under conditions of partial reproducibility varied the day and the analysts to reproduce the variations.

Table 3 and Table 4 show the linearity, recovery, RSD, expanded measurement uncertainty (U), LOD, and LOQ results for analytes determined using LC-MS/MS and GC-MS/MS, respectively. The LOD and LOQ values for 119 analytes determined by LC-MS/MS were 0.0001 mg/kg and 0.0002 mg/kg, respectively, whereas seven analytes showed LOD and LOQ values of 0.0002 mg/kg and 0.0004 mg/kg, and the values for one analyte were 0.0004 mg/kg and 0.0008 mg/kg. For GC-MS/MS analyses, the LOD and LOQ values were 0.001 mg/kg and 0.002 mg/kg for nine analytes, 0.002 mg/kg and 0.004 mg/kg for 30 analytes, and 0.004.0 mg/kg and 0.008 mg/kg for two analytes.

A total of 41 analytes could not be validated, 32 of which were analyzed by LC-MS/MS and 9 by GC-MS/MS (Supplementary Materials Tables S2 and S3). These compounds were detected, but the obtained values for linearity, recovery rate, RSD, and U were not following the European Union SANTE/12682/2019 [34] and Codex Alimentarius CXG90-2017 [35] guidelines.

Pacífico da Silva et al. [1] developed an analytical method with an LC-MS/MS system for the simultaneous detection of 152 pesticides in honey after extraction with ethyl acetate and cleanup using Florisil. The LOD and LOQ values for all the tested pesticides were 0.005 and 0.01 mg/kg, respectively [1]. Paoloni et al. [40] used Florisil for sample cleanup after extraction with n-Hexane for determining 13 pesticides in honey using GC-MS/MS. The LOQ for all tested pesticides was 0.01 mg/kg, and the LOD was not provided [40]. Česnik et al. [31] used a GC-MS method for detecting 75 pesticides and an LC-MS/MS method for detecting 60 pesticides in honey after extraction with a mixture of petroleum ether and dichloromethane. The LOQ ranged from 0.01 to 0.05 mg/kg with the GC-MS method and from 0.003 to 0.01 mg/kg with the LC-MS/MS method [31].

The QuEChERS method was applied for pesticide extraction in honey by other authors [26,27,28,29,30,41,42]. The LC-MS/MS method described by Souza Tette et al. [28] was validated to measure 116 pesticides in honey, but 11 compounds showed recoveries at 0.010 mg/kg out of the 70–120% range. The LOD was 0.005 mg/kg and the LOQ varied between 0.01 and 0.025 mg/kg [28]. The LC-ESI-MS/MS method of Kasiotis et al. [26] detected 115 pesticides, but some analytes showed recoveries below 70%. The LOD ranged from 0.00003 to 0.0233 mg/kg, and the LOQ ranged from 0.0001 to 0.078 mg/kg [26]. Another LC-MS/MS method for analyzing honey samples was described for 207 pesticides [30], with LOQ values ranging from 0.001 to 0.01 mg/kg. However, the LOD was not reported, and some pesticides showed recoveries out of the 70–120% range [30]. In another LC-MS/MS method [29], 132 tested compounds were measured in honey, obtaining recoveries ranging from 70% to 120% for 116 compounds. However, the LOD and LOQ were not provided in the manuscript nor supplementary material [29]. The GC-MS/MS method described by Zheng et al. [41] was validated to measure six pesticides in honey. The LOD ranged from 0.0004 to 0.002 mg/kg and the LOQ varied between 0.001 and 0.005 mg/kg [41]. Another GC-MS/MS method was developed by Shendy et al. [27] for the detection of 200 pesticides in honey. The LOD ranged from 0.001 to 0.003 mg/kg and the LOQ was 0.005 to 0.01 mg/kg, but the recoveries ranged from 51.13–126.55% [27]. Both LC-MS/MS and GC-MS/MS analysis of residual pesticides in honey was described by Bargańska et al. [42]. This method was validated for 51 compounds, 18 of them determined by LC-MS/MS, 21 compounds by GC-MS/MS, and 12 compounds by both methods. The LOD ranged from 0.0028 to 0.09 mg/kg with the LC method and from 0.0023 to 0.027 mg/kg with the GC method [42]. Compared with these above articles, the method described in the present study was able to detect extensive and broad-spectrum pesticides (168) with very high sensitivity.

### 3.3. Real Samples

Of the 33 honey samples analyzed, 31 (93.9%) showed residual levels of pesticides (Table 5). Each sample contained up to 15 detected analytes. The most frequently detected compounds were carbendazim (20 samples), thiabendazole (20 samples), azoxystrobin (15 samples), chlorpyrifos (12 samples), and imidacloprid (12 samples). Carbendazim is a fungicide that is widely used in agriculture. Its toxic effects include liver damage, disruption of endocrine and hematological functions, and reproductive toxicity [43]. Thiabendazole is a fungicide and anthelmintic compound with hepatotoxic and teratogenic effects, and it is probably a carcinogen [44]. Azoxystrobin is also a fungicide, and its toxicity includes lesions in the liver and kidneys [45]. Chlorpyrifos is an organophosphate pesticide that is used as an insecticide and acaricide. It is considered moderately toxic and can cause disruption of neuronal, reproductive, immune, and endocrine systems, cancer, and chromosome damage [46]. Imidacloprid is a neonicotinoid insecticide that is highly toxic to honeybees [1,2], with neurotoxic, immunotoxic, teratogenic, and mutagenic effects in mammals [47]. The presence of pesticides in a considerable percentage of the analyzed samples is indicative of widespread environmental contamination by these compounds. However, the consumption of the analyzed honey may not be considered unsafe because the residual levels of all detected pesticides were below the MRLs established for Brazil [9] and the European Union [6,7,8].

Few studies have been aimed at determining the presence of residual pesticides in honey in Brazil. Organophosphorus trichlorfon was detected in just one sample from one hundred commercial honey samples from five states of Brazil [28]. A total of 19 pesticides were found in 53 honey samples collected directly from colonies in the Rio Grande do Norte state, northeastern Brazil. Thirteen of these pesticides were detected in honey produced by honeybees pollinating melon crops (23 samples); however, only six were found in honey from honeybees foraging in the forest (20 samples), and four in honey produced by the stingless bee *Melipona subnitida* (10 samples) [1]. In another study, honey produced by *M. subnitida* from the Rio Grande do Norte state was tested for residual pesticides. Of the 35 analyzed samples, 25 showed residual pesticides, and the detected compounds were chlorpyrifos-methyl, monocrotophos, and trichlorfon [3]. These data support the requirement for testing honey for the presence of pesticides to avoid commercialization of batches containing residual levels above the MRLs.

## 4. Conclusions

The proposed method was successfully optimized and validated for multi-residue identification and quantification of pesticides in honey. It was able to detect an extensive and broad range of pesticides with remarkably high sensitivity and precision. The developed method was successfully applied to Brazilian commercial honey, showing the analyzed honey was considered safe for consumption.

## Figures and Tables

**Table 1:** Chromatographic parameters and MS/MS (tandem mass spectrometric) detection for compounds analyzed by LC-MS/MS (liquid chromatography tandem mass spectrometric detection).

Name	RT ^1^ (min)	DRT ^2^ (min)	Polarity	Transitions	Collision Energy
2,4-D	9.60	1.5	ESI−	QI ^3^ 218.9 > 161.	11
				1^st^ CI ^4^ 218.9 > 124.9	35
				2^nd^ CI 220.9 > 162.9	11
Acephate	4.56	1.5	ESI+	QI 184.0 > 143.0	5
				1^st^ CI 184.0 > 125.0	15
Acetamiprid	6.56	1.0	ESI+	QI 223.1 > 126.0	15
				1^st^ CI 223.1 > 56.0	15
Aldicarb	8.05	1.5	ESI+	QI 208.1 > 116.2	10
				1^st^ CI 208.1 > 89.1	24
Aldicarb-Sulfone	4.99	1.5	ESI+	QI 223.1 > 148.0	5
				1^st^ CI 223.1 > 76.0	5
Aldicarb-Sulfoxide	4.88	1.5	ESI+	QI 207.1 > 131.9	0
				1^st^ CI 207.1 > 89.1	8
Allethrin	19.36	1.5	ESI+	QI 303.2 > 135.0	10
				1^st^ CI 303.2 > 123	20
Ametryn	13.15	1.5	ESI+	QI 228.1 > 186.1	20
				1^st^ CI 228.1 > 116.1	28
				2^nd^ CI 228.1 > 96.0	25
Aminocarb	7.32	1.5	ESI+	QI 209.1 > 152.2	12
				1^st^ CI 209.1 > 137.2	20
Atrazine	11.34	1.5	ESI+	QI 216.1 > 174.1	16
				1^st^ CI 216.1 > 68.0	40
Avermectin B1a	21.71	1.5	ESI+	QI 890.5 > 567.4	8
				1^st^ CI 890.5 > 305.1	16
Azaconazole	11.03	1.5	ESI+	QI 300.0 > 231.1	16
				1^st^ CI 300.0 > 159.0	28
Azinphos-Ethyl	14.74	1.5	ESI+	QI 346.1 > 132.1	12
				1^st^ CI 346.1 > 97.0	32
Azinphos-Methyl	12.15	1.5	ESI+	QI 318.0 > 261.0	0
				1^st^ CI 318.0 > 132.1	8
Azoxystrobin	12.90	1.5	ESI+	QI 404.1 > 372.1	12
				1^st^ CI 404.1 > 344.1	28
				2^nd^ CI 404.1 > 329.1	36
Benalaxyl	16.86	1.5	ESI+	QI 326.2 > 294.1	4
				1^st^ CI 326.2 > 148.1	27
Bitertanol	16.98	1.5	ESI+	QI 338.2 > 99.1	10
				1^st^ CI 338.2 > 70.0	4
Boscalid	13.34	1.5	ESI+	QI 343.0 > 307.1	16
				1^st^ CI 343.0 > 271.2	32
Bromacil	9.29	1.5	ESI+	QI 261.0 > 205.0	20
				1^st^ CI 261.0 > 187.9	40
Bromuconazole	15.50	3.0	ESI+	QI 378.0 > 159.0	32
				1^st^ CI 378.0 > 70.0	35
Buprofezin	19.13	1.5	ESI+	QI 306.2 > 201.1	5
				1^st^ CI 306.2 > 116.1	10
Cadusafos	17.96	1.5	ESI+	QI 271.1 > 130.9	20
				1^st^ CI 271.1 > 97.0	40
Carbaryl	9.80	1.5	ESI+	QI 202.1 > 145.1	4
				1^st^ CI 202.1 > 127.1	28
Carbendazim	7.07	1.5	ESI+	QI 192.1 > 160.1	16
				1^st^ CI 192.1 > 132.1	32
Carbofuran	9.35	1.5	ESI+	QI 222.1 > 165.1	20
				1^st^ CI 222.1 > 123.1	30
3-Hydroxycarbofuran	6.35	1.5	ESI+	QI 238.1 > 220.1	0
				1^st^ CI 238.1 > 163.1	8
Carboxin	9.88	1.5	ESI+	QI 236.1 > 143.1	12
				1^st^ CI 236.1 > 93.1	36
Chlorfenvinphos	17.20	1.5	ESI+	QI 358.9 > 155.0	8
				1^st^ CI 358.9 > 99.2	28
Chlorfluazuron	20.00	1.5	ESI+	QI 539.9 > 383.0	44
				1^st^ CI 539.9 > 158.0	36
Chlorpyrifos	20.20	1.5	ESI+	QI 349.9 > 198.0	20
				1^st^ CI 349.9 > 97.0	20
Chlorpyrifos-Methyl-Oxon	15.89	1.5	ESI+	QI 334.0 > 306.0	8
				1^st^ CI 334.0 > 278.0	8
Clofentezine	16.99	1.5	ESI+	QI 303.0 > 138.0	12
				1^st^ CI 303.0 > 102.0	40
Clomazone	12.70	1.5	ESI+	QI 242.1 > 127.0	20
				1^st^ CI 240.1 > 125.0	20
				2^nd^ CI 240.1 > 89.1	56
Clothianidin	6.08	1.5	ESI+	QI 250.0 > 169.0	8
				1^st^ CI 250.0 > 131.9	8
Cyanazine	8.43	1.5	ESI+	QI 241.1 > 214.1	18
				1^st^ CI 241.1 > 104.0	44
Cyanofenphos	16.60	1.0	ESI+	QI 304.1 > 276.0	12
				1^st^ CI 304.1 > 157.0	24
Cyazofamid	15.43	1.5	ESI+	QI 325.0 > 261.0	4
				1^st^ CI 325.0 > 108.0	8
Cymoxanil	6.97	1.5	ESI+	QI 199.1 > 128.0	4
				1^st^ CI 199.1 > 110.9	12
Cyproconazole	14.80	2.0	ESI+	QI 292.1 > 125.0	32
				1^st^ CI 292.1 > 70.0	16
Cyprodinil	17.10	1.5	ESI+	QI 226.1 > 108.0	30
				1^st^ CI 226.1 > 93.0	40
Cyromazine	4.48	1.5	ESI+	QI 167.1 > 125.0	16
				1^st^ CI 167.1 > 85.0	16
Diafenthiuron	20.82	1.5	ESI+	QI 385.2 > 329.2	16
				1^st^ CI 385.2 > 278.2	32
Diazinon	17.10	1.5	ESI+	QI 305.1 > 169.1	32
				1^st^ CI 305.1 > 97.0	40
Dichlorvos	9.11	1.5	ESI+	QI 221.0 > 109.0	12
				1^st^ CI 221.0 > 79.0	24
Dicrotophos	5.83	1.5	ESI+	QI 238.0 > 127.0	12
				1^st^ CI 238.0 > 112.1	8
Difenoconazole	17.80	1.5	ESI+	QI 406.1 > 337.0	18
				1^st^ CI 406.1 > 251.0	28
Diflubenzuron	14.96	1.5	ESI+	QI 311.0 > 158.0	8
				1^st^ CI 311.0 > 141.0	32
Dimethoate	6.53	1.5	ESI+	QI 230.0 > 198.8	0
				1^st^ CI 230.0 > 125.0	16
Dimethomorph	13.80	3.0	ESI+	QI 388.1 > 301.1	20
				1^st^ CI 388.1 > 165.1	32
Diniconazole	18.00	1.5	ESI+	QI 326.1 > 159.0	28
				1^st^ CI 326.1 > 70.0	28
Disulfoton	17.69	1.5	ESI+	QI 275.0 > 89.0	12
				1^st^ CI 275.0 > 61.0	44
Disulfoton-Sulfone	10.72	1.5	ESI+	QI 307.0 > 125.0	10
				1^st^ CI 307.0 > 97.0	30
Disulfoton-Sulfoxide	10.78	1.5	ESI+	QI 291.0 > 185.0	10
				1^st^ CI 291.0 > 157.0	20
Diuron	11.38	1.5	ESI+	QI 2350. > 72.0	20
				1^st^ CI 233.0 > 160.0	24
				2^nd^ CI 233.03 > 72.1	20
Emamectin B1a	20.89	2.0	ESI+	QI 886.5 > 158.0	44
				1^st^ CI 886.5 > 82.1	64
Emamectin B1b	20.34	2.0	ESI+	QI 872.5 > 158.3	40
				1^st^ CI 872.5 > 82.3	68
Epoxiconazole	15.21	1.5	ESI+	QI 330.1 > 121.0	16
				1^st^ CI 330.1 > 101.2	52
Ethion	19.65	1.5	ESI+	QI 385.0 > 199.1	4
				1^st^ CI 385.0 > 142.8	24
Etofenprox	22.82	1.5	ESI+	QI 394.2 > 359.0	5
				1^st^ CI 394.2 > 177.0	5
Ethoprophos	15.72	1.5	ESI+	QI 243.1 > 130.9	15
				1^st^ CI 243.1 > 97.0	30
Etrimfos	16.80	1.5	ESI+	QI 293.1 > 265.0	26
				1^st^ CI 293.1 > 125.0	28
Famoxadone	16.93	1.5	ESI+	QI 392.1 > 330.9	4
				1^st^ CI 392.1 > 238.0	12
Fenamiphos	15.92	1.5	ESI+	QI 304.1 > 234.0	12
				1^st^ CI 304.1 > 217.1	20
Fenbuconazole	15.11	1.5	ESI+	QI 337.1 > 125.1	40
				1^st^ CI 337.1 > 70.0	33
Fenpyroximate	20.91	1.5	ESI+	QI 422.2 > 366.2	12
				1^st^ CI 422.2 > 135.0	36
Fenthion	16.86	1.5	ESI+	QI 279.0 > 247.1	8
				1^st^ CI 279.0 > 169.1	12
Fipronil	15.50	1.5	ESI+	QI 437.0 > 368.0	18
				1^st^ CI 437.0 > 255.0	26
Flazasulfuron	11.24	1.5	ESI+	QI 408.1 > 182.1	28
				1^st^ CI 408.1 > 83.0	40
Fluazifop-Butyl	18.80	1.5	ESI+	QI 384.1 > 328.1	12
				1^st^ CI 384.1 > 282.2	20
Flufenoxuron	19.88	1.5	ESI+	QI 489.1 > 158.0	20
				1^st^ CI 489.1 > 140.9	56
Fluquinconazole	15.11	1.5	ESI+	QI 376.0 > 349.0	20
				1^st^ CI 376.0 > 307.1	24
Flutriafol	11.17	1.5	ESI+	QI 302.1 > 122.9	28
				1^st^ CI 302.1 > 70.1	16
Furathiocarb	19.30	1.5	ESI+	QI 383.2 > 251.9	8
				1^st^ CI 383.2 > 195.0	16
Heptenophos	11.79	1.5	ESI+	QI 251.0 > 127.0	15
				1^st^ CI 251.0 > 125.0	25
Hexaconazole	17.40	1.5	ESI+	QI 314.1 > 159.0	30
				1^st^ CI 314.1 > 70.1	20
Hexythiazox	19.90	1.5	ESI+	QI 353.1 > 227.9	8
				1^st^ CI 353.1 > 168.1	24
Imazalil	14.30	3.0	ESI+	QI 297.1 > 201.0	15
				1^st^ CI 297.1 > 159.0	20
Imazapyr	5.48	3.0	ESI+	QI 262.1 > 217.1	20
				1^st^ CI 262.1 > 131.0	40
Imazethapyr	7.42	1.5	ESI+	QI 290.1 > 245.1	24
				1^st^ CI 290.1 > 177.0	29
Imibenconazole	19.60	1.5	ESI+	QI 411.0 > 171.0	20
				1^st^ CI 411.0 > 125.0	40
Imidacloprid	5.97	1.5	ESI+	QI 258.0 > 210.9	12
				1^st^ CI 256.0 > 208.9	12
				2^nd^ CI 256 > 175	12
Indoxacarb	17.88	1.5	ESI+	QI 528.1 > 203.0	45
				1^st^ CI 528.1 > 150.0	20
Iprodione	15.98	1.5	ESI+	QI 330.0 > 287.9	10
				1^st^ CI 330.0 > 244.9	14
Iprovalicarb	15.20	1.5	ESI+	QI 321.2 > 202.9	5
				1^st^ CI 321.2 > 119	16
Kresoxim-Methyl	16.50	1.5	ESI+	QI 314.1 > 267.0	0
				1^st^ CI 314.1 > 222.1	10
Linuron	12.67	1.5	ESI+	QI 249.0 > 160.1	20
				1^st^ CI 249.0 > 133.0	36
Lufenuron	19.03	1.5	ESI+	QI 510.9 > 158.0	20
				1^st^ CI 510.9 > 141.0	57
Malaoxon	9.37	1.5	ESI+	QI 315.0 > 127.1	20
				1^st^ CI 315.0 > 99.2	4
Malathion	14.30	1.5	ESI+	QI 331.0 > 126.9	5
				1^st^ CI 331.0 > 99.0	10
Metalaxyl	11.93	1.5	ESI+	QI 280.2 > 220.1	10
				1^st^ CI 280.2 > 160.1	20
Metconazole	17.30	1.5	ESI+	QI 320.1 > 125.0	48
				1^st^ CI 320.1 > 70.1	24
Methamidophos	4.31	1.5	ESI+	QI 142.0 > 125.0	10
				1^st^ CI 142.0 > 94.0	10
Methidathion	11.95	1.5	ESI+	QI 302.9 > 145.0	0
				1^st^ CI 302.9 > 85.1	15
Methiocarb	13.14	1.5	ESI+	QI 226.1 > 169.0	4
				1^st^ CI 226.1 > 121.1	12
Methomyl	5.44	1.5	ESI+	QI 163.1 > 106.0	4
				1^st^ CI 163.1 > 88.0	0
Methoxyfenozide	14.08	1.5	ESI+	QI 369.2 > 313.1	0
				1^st^ CI 369.2 > 149.0	10
Metolachlor	16.30	1.5	ESI+	QI 284.1 > 252.1	8
				1^st^ CI 284.1 > 176.1	24
Metribuzin	9.38	1.5	ESI+	QI 215.1 > 187.1	15
				1^st^ CI 215.1 > 84.0	30
Mevinphos	7.35	3.0	ESI+	QI 225.0 > 193.1	0
				1^st^ CI 225.0 > 127.0	12
Monocrotophos	5.52	1.5	ESI+	QI 224.1 > 193.0	0
				1^st^ CI 224.1 > 127.0	10
Myclobutanil	14.00	1.5	ESI+	QI 289.1 > 125.1	32
				1^st^ CI 289.1 > 70.1	16
Naled	11.80	1.5	ESI+	QI 380.7 > 127.0	8
				1^st^ CI 380.7 > 109.0	24
Omethoate	4.77	1.5	ESI+	QI 214.0 > 125.0	16
				1^st^ CI 214.0 > 109.0	24
Oxamyl	5.09	1.5	ESI+	QI 237.1 > 90.0	10
				1^st^ CI 237.1 > 72.0	12
Paclobutrazol	13.95	1.2	ESI+	QI 294.1 > 125.2	40
				1^st^ CI 294.1 > 70.1	20
Paraoxon	10.88	1.5	ESI+	QI 276.1 > 220.0	10
				1^st^ CI 276.1 > 94.0	40
Paraoxon-Methyl	8.05	1.5	ESI+	QI 248.0 > 201.9	20
				1^st^ CI 248.0 > 90.0	25
Parathion	16.40	1.5	ESI+	QI 292.0 > 236.1	8
				1^st^ CI 292.0 > 94.1	40
Penconazole	16.70	1.5	ESI+	QI 284.1 > 159	30
				1^st^ CI 284.1 > 70.1	15
Pencycuron	18.00	1.5	ESI+	QI 329.1 > 125.1	24
				1^st^ CI 329.1 > 89.1	60
Pendimethalin	20.20	1.5	ESI+	QI 282.1 > 212.1	4
				1^st^ CI 282.1 > 194.1	16
Phenthoate	16.20	1.5	ESI+	QI 321.0 > 163.1	8
				1^st^ CI 321.0 > 79.1	44
Phorate	17.50	1.5	ESI+	QI 261.0 > 199.0	2
				1^st^ CI 261.0 > 75.1	5
Phosmet	12.80	1.5	ESI+	QI 317.9 > 160.0	8
				1^st^ CI 317.9 > 133.0	36
Phosphamidon	8.30	1.5	ESI+	QI 300.0 > 174.1	8
				1^st^ CI 300.0 > 127.1	16
Picloram	4.71	1.5	ESI+	QI 243.0 > 196.8	22
				1^st^ CI 243.0 > 169.8	34
				2^nd^ CI 241 > 222.8	10
Picoxystrobin	16.03	1.5	ESI+	QI 368.1 > 205.2	4
				1^st^ CI 368.1 > 145.0	20
Pirimicarb	11.08	1.5	ESI+	QI 239.1 > 182.1	12
				1^st^ CI 239.1 > 72.1	20
Pirimiphos-Ethyl	19.39	1.5	ESI+	QI 334.1 > 198.1	22
				1^st^ CI 334.1 > 182.1	24
Pirimiphos-Methyl	18.00	1.5	ESI+	QI 306.2 > 164.1	20
				1^st^ CI 306.2 > 108.1	30
Prochloraz	18.03	1.5	ESI+	QI 376.0 > 308.0	4
				1^st^ CI 376.0 > 265.9	12
Profenofos	18.83	1.5	ESI+	QI 374.9 > 347.0	12
				1^st^ CI 374.9 > 304.9	19
Propamocarb	4.72	1.5	ESI+	QI 189.2 > 144.0	8
				1^st^ CI 189.2 > 102.0	12
Propargite	20.26	1.5	ESI+	QI 368.1 > 231.2	0
				1^st^ CI 368.1 > 175.2	8
Propiconazole	17.50	1.5	ESI+	QI 342.1 > 159.0	32
				1^st^ CI 342.1 > 69.1	16
Propoxur	9.33	1.5	ESI+	QI 210.11 > 168.1	5
				1^st^ CI 210.11 > 111.1	8
Pyraclostrobin	17.50	1.5	ESI+	QI 388.11 > 193.8	8
				1^st^ CI 388.11 > 163.1	20
Pyrazophos	17.26	1.5	ESI+	QI 374.1 > 222.1	16
				1^st^ CI 374.1 > 194.1	32
Pyridaben	21.90	1.5	ESI+	QI 365.1 > 309.1	4
				1^st^ CI 365.1 > 147.2	20
Pyridaphenthion	14.36	1.5	ESI+	QI 341.0 > 205.1	10
				1^st^ CI 341.0 > 189.0	20
Pyrimethanil	13.88	1.5	ESI+	QI 200.1 > 106.9	20
				1^st^ CI 200.1 > 82.0	25
Pyriproxyfen	19.90	1.5	ESI+	QI 322.2 > 227.2	12
				1^st^ CI 322.2 > 185.0	20
				2^nd^ CI 322.2 > 96	12
Quinalphos	16.48	1.5	ESI+	QI 299.0 > 163.0	20
				1^st^ CI 299.0 > 147.0	20
Quizalofop-Ethyl	19.10	1.5	ESI+	QI 373.0 > 271.2	24
				1^st^ CI 373.0 > 255.1	36
Simazine	9.45	1.5	ESI+	QI 202.1 > 132.0	22
				1^st^ CI 202.1 > 124.1	26
Spinosyn A	19.87	2.0	ESI+	QI 732.5 > 142.1	28
				1^st^ CI 732.5 > 98.1	60
Spinosyn D	20.78	2.0	ESI+	QI 746.5 > 142.1	35
				1^st^ CI 746.5 > 98.0	55
Spirodiclofen	21.25	1.5	ESI+	QI 411.1 > 313	8
				1^st^ CI 411.1 > 71.2	15
Spiromesifen	20.67	1.5	ESI+	QI 371.2 > 273.1	12
				1^st^ CI 371.2 > 255.1	24
Sulfentrazone	9.38	1.5	ESI+	QI 404.0 > 306.9	28
				1^st^ CI 404.0 > 273.0	40
Tebuconazole	16.80	1.5	ESI+	QI 308.1 > 124.9	47
				1^st^ CI 308.1 > 70.0	40
Tebufenozide	16.08	1.5	ESI+	QI 353.2 > 297.1	4
				1^st^ CI 353.2 > 133.0	20
Teflubenzuron	19.00	1.5	ESI+	QI 381.0 > 158.0	12
				1^st^ CI 381.0 > 141.0	48
Temephos	18.90	1.5	ESI+	QI 467.0 > 419.0	20
				1^st^ CI 467 > 124.9	44
Terbufos	19.60	1.5	ESI+	QI 289.1 > 233.0	0
				1^st^ CI 289.1 > 57.1	16
Tetraconazole	15.09	1.5	ESI+	QI 372.0 > 159.0	36
				1^st^ CI 372.0 > 70.0	20
Thiabendazole	8.22	1.5	ESI+	QI 202.0 > 175.0	24
				1^st^ CI 202.0 > 131.0	36
Thiacloprid	7.10	1.5	ESI+	QI 253.0 > 126.0	16
				1^st^ CI 253.0 > 90.0	40
Thiamethoxam	5.42	1.5	ESI+	QI 292.0 > 211.1	8
				1^st^ CI 292.0 > 181.1	20
Thiobencarb	18.03	1.5	ESI+	QI 258.0 > 125.1	25
				1^st^ CI 258.07 > 100.1	5
Thiodicarb	10.59	1.5	ESI+	QI 355.0 > 108.1	8
				1^st^ CI 355.0 > 88.1	8
Thiophanate-Methyl	8.65	1.5	ESI+	QI 343.0 > 151.0	20
				1^st^ CI 343.0 > 93.0	56
Tolylfluanid	15.99	1.5	ESI+	QI 346.9 > 238.1	12
				1^st^ CI 346.9 > 137.0	25
Triadimefon	14.80	1.5	ESI+	QI 294.1 > 197.2	8
				1^st^ CI 294.1 > 69.1	16
Triadimenol	14.70	1.5	ESI+	QI 296.1 > 99.1	16
				1^st^ CI 296.1 > 70.0	12
Triazophos	14.30	1.5	ESI+	QI 314.1 > 162.1	16
				1^st^ CI 314.1 > 119.1	36
Trichlorfon	6.55	1.5	ESI+	QI 256.9 > 221.0	4
				1^st^ CI 256.9 > 109.0	12
Trifloxystrobin	18.35	1.5	ESI+	QI 409.1 > 186.0	12
				1^st^ CI 409.1 > 145.0	52
Triflumizole	18.50	1.5	ESI+	QI 346.1 > 278.0	4
				1^st^ CI 346.1 > 43.1	20
Vamidothion	6.39	1.5	ESI+	QI 288.0 > 146.1	6
				1^st^ CI 288.0 > 58.0	44
Zoxamide	16.96	1.5	ESI+	QI 336.0 > 187.0	16
				1^st^ CI 336.0 > 159.0	44

^1^ RT: retention time. ^2^ DRT: delta retention time. ^3^ QI: quantification ions. ^4^ CI: confirmation ions.

**Table 2:** Chromatographic parameters and MS/MS detection for compounds analyzed by GC-MS/MS (gas chromatography tandem mass spectrometric detection).

Name	RT ^1^ (min)	DRT ^2^ (min)	Quantification Transition	Collision Energy
Alachlor	11.33	1	QI ^3^ 188.1 > 160.1	10
			1^st^ CI ^4^ 188.1 > 130.1	40
Aldrin	12.62	1	QI 263.0 > 193.0	30
			1^st^ CI 298.0 > 263.0	8
Bifenthrin	19.78	2	QI 182.0 > 167.0	12
			1^st^ CI 181.0 > 165.0	25
Bromophos-Methyl	13.06	1	QI 330.9 > 315.9	16
			1^st^ CI 329.0 > 314.0	16
Bromopropylate	19.82	2	QI 341.0 > 185.0	5
			1^st^ CI 341.0 > 183.0	20
Carbophenothion	17.71	1	QI 153.0 > 96.9	10
			1^st^ CI 153.0 > 79.0	30
			2^nd^ CI 157.0 > 75.1	40
Cyfluthrin	24.39	2	QI 162.9 > 127.0	5
			1^st^ CI 226.9 > 77.1	30
Cypermethrin	25.01	2	QI 162.9 > 127.0	5
			1^st^ CI 181.1 > 127.1	35
Clordane Gama (Trans)	14.42	2	QI 272.0 > 237.0	16
			1^st^ CI 375.0 > 266.0	25
Chlorfenapyr	16	1	QI 247.0 > 227.0	15
			1^st^ CI 247.0 > 200.0	25
			2^nd^ CI 247.0 > 197.0	5
Chlorothalonil	10.17	1	QI 265.9 > 230.9	20
			1^st^ CI 263.8 > 229.0	20
			2^nd^ CI 263.8 > 168.0	25
Chlorpyrifos-Methyl	11.14	2	QI 288.0 > 93.0	26
			1^st^ CI 288.0 > 273.0	15
			2^nd^ CI 286.0 > 271.0	16
Chlorthiophos	16.93	1	QI 297.0 > 269.0	14
			1^st^ CI 269.0 > 205.0	16
2,4´-DDD	15.7	1	QI 237.0 > 165.0	20
			1^st^ CI 235.0 > 165.0	20
2,4´-DDE	14.47	2	QI 246.0 > 176.0	30
			1^st^ CI 248.0 > 211.0	20
4,4´-DDE	15.92	1	QI 246.0 > 176.0	30
			1^st^ CI 248.0 > 176.0	20
2,4´-DDT	16.84	1	QI 237.0 > 165.0	20
			1^st^ CI 235.0 > 165.0	20
4,4´-DDT	18	1	QI 237.0 > 165.0	20
			1^st^ CI 235.0 > 165.0	20
Deltamethrin	27.94	1	QI 253.0 > 93.0	20
			1^st^ CI 253.0 > 174.0	15
Dicofol	18.46	2	QI 253.0 > 141.0	15
			1^st^ CI 249.9 > 139.1	10
Dieldrin	15.69	1	QI 263.0 > 191.0	35
			1^st^ CI 263.0 > 193.0	35
Endosulfan Alpha	14.84	2	QI 238.8 > 204.0	15
			1^st^ CI 241.0 > 206.0	15
Endosulfan Beta	16.35	1	QI 241.0 > 206.0	15
			1^st^ CI 195.0 > 159.0	15
Endosulfan Sulfate	17.94	1	QI 271.9 > 236.9	15
			1^st^ CI 240.8 > 205.9	15
Endrin	16.06	1	QI 263.0 > 191.0	35
			1^st^ CI 263.0 > 193.0	35
Esfenvalerate	26.91	2	QI 225.0 > 119.0	15
			1^st^ CI 167.0 > 125.0	10
Fenpropathrin	20.1	1	QI 265.0 > 210.0	15
			1^st^ CI 265.0 > 89.0	35
			2^nd^ CI 181.0 > 152.0	26
Fenarimol	21.94	1	QI 139.0 > 111.0	15
			1^st^ CI 219.0 > 107.0	10
Fenitrothion	11.94	1	QI 277.0 > 260.0	5
			1^st^ CI 277.1 > 109.0	20
			2^nd^ CI 276.8 > 125.0	15
Phosalone	20.91	1	QI 182.0 > 111.0	15
			1^st^ CI 182.0 > 75.1	40
HCH Alpha	9.1	2	QI 180.9 > 145.0	12
			1^st^ CI 218.8 > 183.0	5
HCH Beta	9.57	2	QI 180.9 > 145.0	12
			1^st^ CI 218.8 > 183.0	5
HCH Delta	10.38	1	QI 180.9 > 145.0	12
			1^st^ CI 218.8 > 183.0	5
HCH Gamma	9.81	2	QI 180.9 > 145.0	12
			1^st^ CI 218.8 > 183.0	5
Heptachlor	11.63	1	QI 271.9 > 236.8	25
			1^st^ CI 274.0 > 239.0	20
Heptacloro Exo Epoxid	13.71	1	QI 353.0 > 263.0	15
			1^st^ CI 353.0 > 282.0	15
Hexachlorobenzene (HCB)	9.23	1	QI 283.9 > 213.9	35
			1^st^ CI 283.9 > 248.8	25
Lambda Cyhalothrin	21.65	1	QI 181.1 > 152.1	30
			1^st^ CI 197.0 > 161.0	10
Methoxychlor	20	2	QI 227.0 > 141.1	40
			1^st^ CI 227.0 > 169.0	20
Mirex	21.68	1	QI 271.9 > 235.0	25
			1^st^ CI 272.0 > 237.0	20
Ovex (Clorfenson)	15.11	1	QI 174.8 > 111.1	10
			1^st^ CI 177.0 > 113.0	12
			2^nd^ CI 302.0 > 175.0	4
Oxyfluorfen	15.64	1	QI 252.0 > 146.0	32
			1^st^ CI 252.0 > 170.0	32
Parathion-Methyl	11.28	2	QI 263.0 > 109.1	15
			1^st^ CI 263.0 > 79.1	30
Permethrin	23.38	2	QI 183.1 > 153.1	15
			1^st^ CI 183.0 > 115.2	25
Procymidone	13.97	2	QI 283.0 > 96.0	10
			1^st^ CI 283.0 > 67.1	40
Prothiofos	15.21	1	QI 162.0 > 63.1	40
			1^st^ CI 267.0 > 239.0	5
Quintozene	9.73	1	QI 249.0 > 214.0	20
			1^st^ CI 295.0 > 237.0	20
Tetradifon	20.71	1	QI 226.9 > 199.0	10
			1^st^ CI 355.7 > 159.0	10
Trifluralin	8.48	1	QI 306.0 > 264.0	10
			1^st^ CI 263.9 > 160.1	15
Vinclozolin	11.22	2	QI 212.0 > 172.0	15
			1^st^ CI 212.0 > 109.0	40

^1^ RT: retention time. ^2^ DRT: delta retention time. ^3^ QI: quantification ions. ^4^ CI: confirmation ions.

**Table 3:** Linearity, recovery (in %), repeatability relative standard deviation (RSD; in %), expanded measurement uncertainty (U; in %), limit of detection (LOD; in mg/kg), and limit of quantification (LOQ; in mg/kg) for each analyte of the LC-MS/MS method for analysis of pesticides in honey.

Compound	Linearity			Average Recovery			RSD			U			LOD	LOQ
	Type of Adjust	Ponderation	LR ^1^ (µg/kg)	Pt ^2^ 1	Pt 2	Pt 6	Pt 1	Pt 2	Pt 6	Pt 1	Pt 2	Pt 6	(mg/kg)	(mg/kg)
3-Hydroxycarbofuran	Linear		1–10	110	115	92	6	6	8	6	6	8	0.00010	0.00020
Acephate	Linear	1/x	1–10	109	91	81	4	10	5	8	10	10	0.00010	0.00020
Acetamiprid	Linear	1/x	1–10	101	96	101	10	7	4	20	19	8	0.00010	0.00020
Aldicarb	Linear	1/x	1–10	95	98	86	8	11	12	12	15	12	0.00010	0.00020
Aldicarb-sulfone	Linear		1–10	118	104	90	5	11	6	5	11	6	0.00010	0.00020
Aldicarb-sulfoxide	Linear		1–10	118	111	100	7	11	9	8	14	17	0.00010	0.00020
Allethrin	Linear		1–10	92	80	83	10	18	20	10	18	20	0.00010	0.00020
Ametryn	Linear		1–10	119	106	99	3	4	4	6	4	9	0.00010	0.00020
Aminocarb	Linear	1/x	1–10	97	100	90	13	9	4	14	9	12	0.00010	0.00020
Atrazine	Linear		1–10	107	104	103	4	5	4	14	10	6	0.00010	0.00020
Azaconazole	Linear		1–10	108	107	108	12	10	4	12	11	10	0.00010	0.00020
Azinphos-ethyl	Linear		1–10	104	106	96	8	7	7	8	8	9	0.00010	0.00020
Azinphos-methyl	Linear		1–10	119	98	88	9	3	4	9	14	14	0.00010	0.00020
Azoxystrobin	Linear	1/x	1–10	105	93	99	7	3	4	14	20	16	0.00010	0.00020
Benalaxyl	Linear		1–10	119	109	97	6	7	8	10	7	8	0.00010	0.00020
Bitertanol	Linear		1–10	111	103	94	4	8	5	7	8	5	0.00010	0.00020
Boscalid	Linear		1–10	119	97	88	10	4	6	12	20	14	0.00010	0.00020
Bromacil	Linear	1/x	1–10	100	95	92	6	6	4	16	19	12	0.00010	0.00020
Bromuconazole	Linear		2–20	118	108	101	4	5	4	7	5	4	0.00020	0.00040
Buprofezin	Linear	1/x	1–10	102	103	88	18	17	14	19	17	14	0.00010	0.00020
Cadusafos	Linear		1–10	110	102	92	5	7	11	9	7	11	0.00010	0.00020
Carbaryl	Linear		1–10	116	95	95	10	7	4	10	12	11	0.00010	0.00020
Carbendazim	Linear	1/x	1–10	114	108	114	6	8	3	7	12	12	0.00010	0.00020
Carbofuran	Linear	1/x	1–10	106	99	101	4	2	3	9	18	15	0.00010	0.00020
Chlorfenvinphos	Linear	1/x	1–10	111	110	108	4	6	5	10	6	5	0.00010	0.00020
Chlorpyrifos	Linear		1–10	91	79	74	14	18	16	16	18	16	0.00010	0.00020
Chlorpyrifos-methyl-oxon	Linear		1–10	113	108	100	4	6	9	6	6	9	0.00010	0.00020
Clofentezine	Linear		1–10	117	105	92	6	9	9	9	12	9	0.00010	0.00020
Clomazone	Linear	1/x	1–10	105	88	99	8	5	5	12	19	15	0.00010	0.00020
Clothianidin	Linear		1–10	119	105	100	5	6	6	10	8	6	0.00010	0.00020
Cyanazine	Linear		1–10	118	95	90	9	4	7	9	19	19	0.00010	0.00020
Cyanofenphos	Linear	1/x	1–10	105	102	96	9	15	11	10	15	11	0.00010	0.00020
Cyazofamid	Linear		1–10	105	99	94	7	4	5	19	18	9	0.00010	0.00020
Cyproconazole	Linear	1/x²	2–20	105	110	115	3	4	2	15	7	4	0.00020	0.00040
Cyprodinil	Linear		1–10	100	106	95	7	18	11	13	18	11	0.00020	0.00040
Dicrotophos	Linear	1/x	1–10	104	92	93	5	5	4	13	19	16	0.00010	0.00020
Difenoconazole	Linear		1–10	113	105	100	5	7	8	5	8	9	0.00010	0.00020
Diflubenzuron	Linear		1–10	113	106	101	3	9	10	5	9	10	0.00010	0.00020
Dimethoate	Linear		1–10	100	100	99	8	8	4	10	12	4	0.00010	0.00020
Dimethomorph	Linear		1–10	119	109	102	5	3	5	6	8	5	0.00010	0.00020
Diniconazole	Linear	1/x	1–10	97	101	97	7	8	10	10	12	11	0.00010	0.00020
Disulfoton-sulfone	Linear		1–10	110	109	106	3	4	5	10	8	6	0.00010	0.00020
Diuron	Linear	1/x	1–10	112	104	110	4	10	3	13	11	4	0.00010	0.00020
Emamectin B1a	Linear		1–10	114	118	106	6	7	5	8	8	5	0.00010	0.00020
Emamectin B1b	Linear		1–10	113	112	111	8	9	6	13	10	6	0.00010	0.00020
Epoxiconazole	Linear		1–10	109	110	102	8	5	5	8	6	8	0.00010	0.00020
Ethion	Linear	1/x²	1–10	84	88	87	9	19	20	16	19	20	0.00010	0.00020
Ethoprophos	Linear		1–10	107	101	92	6	7	8	6	7	9	0.00010	0.00020
Etrimfos	Linear	1/x	1–10	106	100	97	7	5	7	8	6	9	0.00010	0.00020
Famoxadone	Linear	1/x	1–10	106	98	88	6	11	12	16	11	12	0.00010	0.00020
Fenbuconazole	Linear	1/x	1–10	98	100	108	5	6	5	20	17	8	0.00010	0.00020
Fenpyroximate	Linear		1–10	113	103	95	6	9	4	6	13	20	0.00010	0.00020
Fenthion	Linear	1/x	1–10	106	94	90	5	10	8	19	12	11	0.00010	0.00020
Fipronil	Linear	1/x²	2–20	106	101	99	5	11	8	6	11	8	0.00020	0.00040
Fluazifop-P-butyl	Linear	1/x	1–10	106	87	77	8	12	16	9	12	16	0.00010	0.00020
Fluquinconazole	Linear		1–10	106	106	110	7	6	6	10	8	6	0.00010	0.00020
Furathiocarb	Linear		1–10	107	96	88	4	6	10	6	6	10	0.00010	0.00020
Heptenophos	Linear		1–10	104	97	94	7	7	8	8	9	12	0.00010	0.00020
Hexaconazole	Linear	1/x	1–10	105	108	100	6	9	6	14	11	9	0.00010	0.00020
Hexythiazox	Linear		1–10	97	87	77	5	16	15	20	16	15	0.00010	0.00020
Imazalil	Linear	1/x	1–10	106	102	103	9	4	6	17	16	8	0.00010	0.00020
Imibenconazole	Linear	1/x	1–10	89.5	85.8	79.9	4.8	9.9	13.6	20.0	12.9	13.6	0.00010	0.00020
Imidacloprid	Linear		1–10	99	91	92	10	10	4	10	18	18	0.00010	0.00020
Indoxacarb	Linear		1–10	105	95	87	5	11	11	6	11	11	0.00010	0.00020
Iprodione	Linear	1/x²	1–10	94	101	95	14	10	15	14	11	16	0.00010	0.00020
Iprovalicarb	Linear		1–10	103	106	105	4	7	4	16	14	7	0.00010	0.00020
Kresoxim-methyl	Linear		1–10	112	104	96	6	6	4	6	9	8	0.00010	0.00020
Linuron	Linear		1–10	117	91	90	18	11	3	18	19	10	0.00010	0.00020
Malaoxon	Linear		1–10	118	117	100	19	10	7	19	11	8	0.00010	0.00020
Malathion	Linear		1–10	102	104	109	4	3	4	20	19	16	0.00010	0.00020
Metalaxyl	Linear	1/x	1–10	108	105	107	2	4	4	14	14	11	0.00010	0.00020
Metconazole	Linear		1–10	111	106	94	4	7	9	5	7	9	0.00010	0.00020
Methidathion	Linear	1/x	1–10	108	100	96	9	4	5	10	12	11	0.00010	0.00020
Methiocarb	Linear		1–10	120	100	91	7	6	3	10	20	19	0.00010	0.00020
Methomyl	Linear	1/x	1–10	92	85	88	10	10	6	19	20	20	0.00010	0.00020
Methoxyfenozide	Linear		1–10	110	108	99	3	6	7	6	6	7	0.00010	0.00020
Metolachlor	Linear	1/x	1–10	105	105	100	5	4	9	20	12	9	0.00010	0.00020
Metribuzin	Linear	1/x	1–10	96	97	98	15	9	5	20	10	12	0.00010	0.00020
Monocrotophos	Linear		1–10	108	90	82	13	15	6	15	16	17	0.00010	0.00020
Myclobutanil	Linear		1–10	110	108	103	5	3	5	11	5	5	0.00010	0.00020
Omethoate	Linear		1–10	95	84	78	7	5	3	8	10	6	0.00010	0.00020
Oxamyl	Linear		1–10	99	101	100	10	10	3	19	17	10	0.00010	0.00020
Paclobutrazol	Linear		1–10	107	98	97	6	3	3	10	9	10	0.00010	0.00020
Paraoxon	Linear		1–10	111	119	117	10	8	5	10	15	15	0.00010	0.00020
Parathion	Linear	1/x	4–40	94	99	93	16	11	14	16	11	14	0.00040	0.00080
Penconazole	Linear		1–10	107	113	103	8	6	4	10	8	6	0.00010	0.00020
Pencycuron	Linear	1/x	1–10	92	86	89	7	12	15	12	12	15	0.00010	0.00020
Pendimethalin	Linear		1–10	98	82	80	12	15	13	14	15	13	0.00010	0.00020
Phenthoate	Linear		1–10	109	101	93	5	10	12	7	10	15	0.00010	0.00020
Phosmet	Linear		1–10	107	104	108	5	4	4	14	16	10	0.00010	0.00020
Phosphamidon	Linear		1–10	118	108	100	4	3	4	7	6	5	0.00010	0.00020
Picoxystrobin	Linear		1–10	110	107	110	6	5	7	15	9	7	0.00010	0.00020
Pirimicarb	Linear	1/x	1–10	115	110	111	3	2	4	9	4	4	0.00010	0.00020
Pirimiphos-ethyl	Linear		1-10	102	90	89	9	8	13	16	8	13	0.00010	0.00020
Pirimiphos-methyl	Linear	1/x	1–10	104	101	95	7	10	11	10	14	13	0.00010	0.00020
Prochloraz	Linear	1/x	1–10	108	106	106	3	7	6	12	10	11	0.00010	0.00020
Profenofos	Linear	1/x²	2–20	103	98	95	5	10	10	10	10	12	0.00020	0.00040
Propargite	Linear		1–10	104	88	73	12	18	19	12	18	19	0.00010	0.00020
Propoxur	Linear	1/x	1–10	108	98	95	10	4	5	15	20	19	0.00010	0.00020
Pyraclostrobin	Linear		1–10	110	99	96	7	10	11	10	12	13	0.00010	0.00020
Pyrazophos	Linear		1–10	111	104	96	4	7	7	6	7	7	0.00010	0.00020
Pyridaphenthion	Linear		1–10	117	111	102	5	6	4	6	6	4	0.00010	0.00020
Pyriproxyfen	Quadratic	1/x²	1–10	98	92	93	18	15	17	20	15	17	0.00010	0.00020
Quinalphos	Linear		1–10	110	105	98	5	7	8	6	7	10	0.00010	0.00020
Quizalofop-P-ethyl	Linear		1–10	96	88	82	8	10	15	8	10	17	0.00010	0.00020
Simazine	Linear	1/x	1–10	104	103	96	5	6	5	15	18	14	0.00010	0.00020
Spinosyn A	Linear		1–10	105	106	101	4	5	6	5	5	6	0.00010	0.00020
Spinosyn D	Linear		1–10	106	101	97	13	17	14	17	17	16	0.00010	0.00020
Spirodiclofen	Linear		1–10	99	84	84	11	19	16	11	19	20	0.00010	0.00020
Spiromesifen	Linear		1–10	86	81	73	10	12	17	10	12	17	0.00010	0.00020
Tebuconazole	Linear		1–10	96	102	100	7	4	8	12	9	8	0.00010	0.00020
Tebufenozide	Linear		1–10	106	100	84	11	10	16	11	10	16	0.00010	0.00020
Teflubenzuron	Linear	1/x	2–20	104	90	81	9	13	18	10	13	18	0.00020	0.00040
Terbufos	Linear	1/x²	2–20	86	74	71	13	12	11	19	13	11	0.00020	0.00040
Tetraconazole	Linear	1/x	1–10	102	105	105	5	6	6	9	11	10	0.00010	0.00020
Thiabendazole	Linear	1/x	1–10	111	114	95	17	18	5	19	18	5	0.00010	0.00020
Thiacloprid	Linear		1–10	117	103	98	6	5	3	6	6	3	0.00010	0.00020
Thiamethoxam	Linear	1/x	1–10	106	94	91	8	7	6	8	9	8	0.00010	0.00020
Thiobencarb	Linear	1/x	1–10	106	94	91	11	7	8	13	9	9	0.00010	0.00020
Thiodicarb	Linear		1–10	111	98	97	10	3	4	10	14	9	0.00010	0.00020
Tolylfluanid	Linear		1–10	100	95	96	8	5	7	12	8	8	0.00010	0.00020
Triadimefon	Linear	1/x	1–10	103	106	104	6	8	5	14	13	11	0.00010	0.00020
Triadimenol	Linear		1–10	116	116	106	4	3	5	6	9	11	0.00010	0.00020
Triazophos	Linear	1/x	1–10	113	104	109	4	5	3	14	13	11	0.00010	0.00020
Trichlorfon	Linear	1/x²	1–10	107	101	97	9	7	5	9	9	9	0.00010	0.00020
Trifloxystrobin	Linear		1–10	105	96	93	5	11	12	9	11	12	0.00010	0.00020
Triflumizole	Linear		1–10	93	94	92	9	7	13	10	7	13	0.00010	0.00020
Vamidothion	Linear	1/x	1–10	105	92	90	6	7	4	12	15	6	0.00010	0.00020
Zoxamide	Linear		1–10	104	92	88	6	9	8	9	10	10	0.00010	0.00020

^1^ LR: linearity range. ^2^ Pt: point.

**Table 4:** Linearity, recovery (in %), repeatability relative standard deviation (RSD; in %), expanded measurement uncertainty (U; in %), limit of detection (LOD; in mg/kg), and limit of quantification (LO Q; in mg/kg) for each analyte of the GC-MS/MS method for analysis of pesticides in honey.

Compound	Linearity			Average Recovery		RSD		U		LOD	LOQ
	Type of Adjust	Ponderation	FT (µg/kg)	Pt 1	Pt 6	Pt 1	Pt 6	Pt 1	Pt 6	(mg/kg)	(mg/kg)
DDE 4,4	Linear	1/x	10–100	119	94	8	7	20	7	0.001	0.002
Alachlor	Linear		10–100	103	109	7	5	19	12	0.001	0.002
Aldrin	Linear	1/x	20–200	110	99	10	9	18	11	0.002	0.004
Azoxystrobin	Linear		10–100	98	78	9	7	13	8	0.001	0.002
Bifenthrin	Linear		20–200	117	91	3	4	11	4	0.002	0.004
Bromophos-methyl	Linear		20–200	119	100	6	7	15	16	0.002	0.004
Bromopropylate	Linear	1/x	20–200	113	91	6	6	14	7	0.002	0.004
Carbophenothion	Linear	1/x	20–200	115	93	5	5	17	5	0.002	0.004
Cyfluthrin	Linear	1/x	40–400	106	92	5	8	13	8	0.004	0.008
Cypermethrin	Linear		20–200	102	89	7	8	16	8	0.002	0.004
Clordane gama (trans)	Linear	1/x	20–200	112	99	7	5	18	10	0.002	0.004
Chlorfenapyr	Linear	1/x	20–200	103	101	8	5	20	6	0.002	0.004
Chlorfenvinphos	Linear		10–100	120	98	10	4	16	13	0.001	0.002
Chlorpyrifos-methyl	Linear	1/x	10–100	113	101	15	9	20	18	0.001	0.002
Chlorthiophos	Linear	1/x	20–200	118	94	9	6	18	6	0.002	0.004
DDD 2,4	Linear	1/x	10–100	115	95	10	7	14	7	0.001	0.002
DDT 2,4	Linear	1/x	10–100	112	98	5	7	20	8	0.001	0.002
DDT 4,4	Linear	1/x	20–200	109	98	5	5	19	7	0.002	0.004
Deltamethrin	Linear	1/x²	10–100	96	119	13	4	20	10	0.001	0.002
Dieldrin	Linear	1/x	20–200	113	96	10	7	20	8	0.002	0.004
Difenoconazole	Linear	1/x	10–100	99	85	9	7	16	8	0.001	0.002
Endosulfan alpha	Linear	1/x	20–200	116	98	8	5	17	8	0.002	0.004
Endosulfan beta	Linear	1/x	20–200	111	95	5	6	18	7	0.002	0.004
Endosulfan sulfate	Linear	1/x	20–200	108	103	7	5	18	6	0.002	0.004
Endrin	Linear	1/x	20–200	111	98	10	5	19	6	0.002	0.004
Esfenvalerate	Linear	1/x	20–200	100	105	8	6	19	9	0.002	0.004
Fenpropathrin	Linear	1/x	20–200	109	92	5	5	16	5	0.002	0.004
Fenarimol	Linear	1/x	20–200	105	85	6	7	12	10	0.002	0.004
Fipronil	Linear	1/x	20–200	109	104	11	4	19	15	0.002	0.004
Fluquinconazole	Linear	1/x	10–100	108	90	7	5	13	6	0.001	0.002
Phosalone	Linear	1/x	20–200	102	92	8	7	18	7	0.002	0.004
HCH alpha	Linear	1/x	20–200	107	84	8	11	13	11	0.002	0.004
Heptachlor	Linear	1/x	20–200	108	90	12	11	18	16	0.002	0.004
Hexachlorobenzene (HCB)	Linear	1/x	10–100	98	80	12	9	13	13	0.001	0.002
Iprodione	Linear	1/x	20–200	108	89	13	8	15	8	0.002	0.004
Lambda cyhalothrin	Linear	1/x	20–200	110	101	7	6	16	6	0.002	0.004
Methoxychlor	Linear	1/x	20–200	108	97	5	6	19	6	0.002	0.004
Mirex	Linear	1/x	10–100	113	108	5	9	18	18	0.001	0.002
Chlorfenson	Linear	1/x	20–200	109	93	9	6	17	8	0.002	0.004
Oxyfluorfen	Linear	1/x²	20–200	108	113	5	6	19	15	0.002	0.004
Pendimethalin	Linear	1/x	10–100	99	104	6	6	18	18	0.001	0.002
Permethrin	Linear	1/x	20–200	99	86	11	5	18	6	0.002	0.004
Pirimicarb	Linear	1/x	10–100	115	104	13	6	14	16	0.001	0.002
Pirimiphos-ethyl	Linear	1/x	10–100	102	105	7	5	18	16	0.001	0.002
Procymidone	Linear	1/x	20–200	103	96	6	7	10	10	0.002	0.004
Profenofos	Linear	1/x	20–200	112	102	4	5	14	13	0.002	0.004
Prothiofos	Linear	1/x	20–200	113	97	8	5	15	8	0.002	0.004
Quintozene	Linear	1/x	10–100	106	87	9	14	18	14	0.001	0.002
Tetradifon	Linear	1/x	40–400	107	90	4	7	15	8	0.004	0.008
Trifluralin	Linear	1/x	20–200	112	94	9	11	15	14	0.002	0.004
Vinclozolin	Linear	1/x	20–200	111	101	6	7	8	14	0.002	0.004

**Table 5:** Detected pesticides (in mg/kg) in 33 samples of honey using the developed LC-MS/MS and GC-MS/MS method.

Compound	Positive Samples	Maximum Levels	LOD ^1^	LOQ ^2^	MRL ^3^
Acephate	8	0.00779	0.0001	0.0002	0.020
Acetamiprid	1	<LQ	0.0001	0.0002	0.050
Azoxystrobin	15	0.00019	0.0001	0.0002	0.050
Bifenthrin	3	<LQ	0.002	0.004	0.010
Boscalid	1	<LQ	0.0001	0.0002	0.050
Carbaryl	2	0.00050	0.0001	0.0002	0.050
Carbendazim	20	0.00350	0.0001	0.0002	1.0
Clomazone	5	<LQ	0.0001	0.0002	-
Chlorpyrifos	12	0.00034	0.0001	0.0002	0.010
Clothianidin	2	0.00063	0.0001	0.0002	-
Diflubenzuron	3	0.00026	0.0001	0.0002	0.050
Dimethoate	6	0.00194	0.0001	0.0002	0.010
Diuron	5	<LQ	0.0001	0.0002	0.050
Imidacloprid	12	0.00618	0.0001	0.0002	0.050
Metoxyphenazide	1	<LQ	0.0001	0.0002	0.050
Omethoate	2	<LQ	0.0001	0.0002	0.010
Pyraclostrobin	2	<LQ	0.0001	0.0002	0.050
Pyrimethanil	3	0.00040	0.0001	0.0002	-
Pyriproxyfen	3	<LQ	0.0001	0.0002	0.050
Tebuconazole	10	0.00045	0.0001	0.0002	0.050
Thiabendazole	20	0.00130	0.0001	0.0002	0.010
Thiamethoxam	9	0.00209	0.0001	0.0002	0.050
Triazophos	1	<LQ	0.0001	0.0002	0.010
Trifloxystrobin	5	0.00030	0.0001	0.0002	0.050

^1^ LOD: limit of detection (in mg/kg). ^2^ LOQ: limit of quantification (in mg/kg). ^3^ MRL: maximum residue level (in mg/kg) [9].

## Supplementary Materials

The following are available online at https://www.mdpi.com/2304-8158/9/10/1368/s1, Table S1. Modified QuEChERS method optimization. Extraction with acetonitrile, or a solution of acetonitrile and ethyl acetate (70:30, v/v), and inclusion of a freezing out step prior to clean up (900 mg of anhydrous magnesium sulfate and 150 mg of PSA). Results are presented as recovery (in %) for each analyte of the LC-MS/MS; Table S2. Non-approved analytes. Linearity, recovery (in %), repeatability relative standard deviation (RSD; in %), expanded measurement uncertainty (U; in %), limit of detection (LOD; in mg/kg), and limit of quantification (LOQ; in mg/kg) for each analyte of the LC-MS/MS method for analysis of pesticides in honey; Table S3: Non-approved analytes. Linearity, recovery (in %), repeatability relative standard deviation (RSD; in %), expanded measurement uncertainty (U; in %), limit of detection (LOD; in mg/kg), and limit of quantification (LOQ; in mg/kg) for each analyte of the GC-MS/MS method for analysis of pesticides in honey.
