# Supplementary material for: Optimization of Method for Pesticide Detection in Honey by Using Liquid and Gas Chromatography Coupled with Mass Spectrometric Detection
Source: Foods. 2020 Sep 26;9(10):1368. doi: 10.3390/foods9101368 (PMC7599512; doi:10.3390/foods9101368)
Supplement: Supplementary file 1 [file foods-09-01368-s001.pdf]

Table S1. Modified QuEChERS method optimization. Extraction with acetonitrile, or a solution of acetonitrile and ethyl acetate (70:30, *v*/*v*), and inclusion of a freezing out step prior to clean up (900 mg of anhydrous magnesium sulfate and 150 mg of PSA). Results are presented as recovery (in %) for each analyte of the LC-MS/MS.

**Analytes**	**Solvents**			
	**Acetonitrile and Ethyl Acetate (70:30, *v*/*v*)**		**Acetonitrile**	
	**No Freeze Out**	**Freeze Out**	**No Freeze Out**	**Freeze Out**
8.9-Z-Avermectin B1A	94	133	114	113
Acephate	71	77	73	83
Acetamiprid	101	106	96	98
Aldicarb	91	99	99	97
Aminocarb	94	101	97	96
Atrazine	94	105	100	100
Bromacil	92	106	92	96
Carbendazim	103	92	85	90
Carbofuran	96	109	103	104
Carbosulfan	104	110	92	90
Carboxin	101	112	102	104
Chlorfenvinphos	85	102	102	101
Chlorpyriphos	91	109	99	106
Clomazone	81	107	101	101
Cyazofamid	96	121	112	110
Cyproconazole	95	109	99	102
Cyprodinil	104	111	99	106
Dicrotophos	94	102	96	95
Dimethoate	95	104	95	100
Diniconazole	94	115	112	112
Disulfoton	87	117	95	5
Disulfoton-Sulfone	100	109	98	100
Diuron	92	105	97	99
Famoxadone	92	102	107	105
Fenbuconazole	88	112	97	103
Fenpyroximate	91	114	100	104
Fenthion	93	107	88	108
Fluazifop-P-butyl	95	113	102	107
Fluquinconazole	99	126	88	104
Flutriafol	91	103	96	99
Hexaconazole	83	94	88	97
Hexythiazox	96	116	97	104
Imazalil	99	112	115	110
Imibenconazole	96	112	115	110
Imidacloprid	107	108	98	77
Iprovalicarb	104	93	94	60
Malathion	92	108	99	107
Metalaxyl	89	106	103	100
Methiocarb	96	109	100	104
Methomyl	95	112	98	99
Metolachlor	98	107	103	106
Metribuzin	92	101	101	109
Mevinphos	104	112	103	106
Naled	16	11	17	7
Oxamyl	94	102	96	95
Paraoxon-methyl	99	112	99	97
Parathion	114	177	99	96
Pencycuron	96	114	102	103
Pendimethalin	96	117	105	109
Phosmet	94	109	95	102
Picoxystrobin	103	115	102	106
Pirimicarb	97	108	100	100
Pirimiphos-ethyl	94	111	103	108
Pirimiphos-methyl	98	115	97	99
Prochloraz	97	108	103	98

Propargite	93	116	98	105
Propiconazole	96	108	109	118
Propoxur	99	108	100	104
Pyraclostrobin	105	114	109	108
Pyrimethanil	102	116	102	110
Simazine	101	103	93	92
Tebuconazole	106	114	108	106
Temephos	96	115	101	107
Terbufos	101	109	103	112
Tetraconazole	99	106	104	113
Thiabendazole	95	102	88	89
Thiamethoxam	95	101	105	103
Thiobencarb	106	113	107	109
Triadimefon	90	114	99	110
Triazophos	99	108	100	104
Trichlorfon	85	84	93	91
Trifloxystrobin	98	112	101	108
Vamidothion	97	106	95	101

Table S2. Non-approved analytes. Linearity, recovery (in %), repeatability relative standard deviation (RSD; in %), expanded measurement uncertainty (U; in %), limit of detection (LOD; in mg/kg), and limit of quantification (LOQ; in mg/kg) for each analyte of the LC-MS/MS method for analysis of pesticides in honey.

Compound	Linearity			Average Recovery			RSD			U			LOD	LOQ
	Type of Adjust	Ponderation	LR[1] (µg/kg)	Pt 1[2]	Pt 2	Pt 6	Pt 1	Pt 2	Pt 6	Pt 1	Pt 2	Pt 6	(mg/kg)	(mg/kg)
2,4-D	Linear	1/x	2-20	6	5	5	24	27	19	54	46	55	0.0002	0.0004
Alachlor	Linear	1/x	1-10	67	94	100	10	12	3	76	26	12	0.0001	0.0002
Avermectin B1a	Linear		1-10	34	66	85	54	25	30	54	25	30	0.0001	0.0002
Bifenthrin	Linear	$1/x^2$	2-20	118	115	85	34	53	50	36	53	57	0.0002	0.0004
Carboxin			1-10	84	67	65	21	19	27	29	21	34	0.0001	0.0002
Chlorfluazuron			1-10	98	96	62	20	43	25	20	43	25	0.0001	0.0002
Cymoxanil			1-10	114	97	94	10	6	3	10	8	7	0.0001	0.0002
Cyromazine			1-10	44	34	17	34	59	18	45	73	18	0.0001	0.0002
Deltamethrin	Linear	$1/x^2$	1-10	67	74	59	40	71	64	106	71	68	0.0001	0.0002
Diafenthiuron			2-20	8	1	0	245	345	346	245	346	346	0.0002	0.0004
Diazinon	Linear	$1/x^2$	1-10	157	163	108	77	14	14	83	32	14	0.0001	0.0002
Dichlorvos	Linear		2-20	76	85	88	41	17	33	41	24	39	0.0002	0.0004
Disulfoton			2-20	71	61	56	15	23	27	15	33	45	0.0002	0.0004
Disulfoton-sulfoxide	Linear		1-10	178	169	170	20	13	20	20	26	36	0.0001	0.0002
Etofenprox			1-10	175	179	133	55	72	55	81	72	55	0.0001	0.0002
Fenamiphos			1-10	101	94	94	7	11	13	9	16	16	0.0001	0.0002
Flazasulfuron	Linear		1-10	28	20	16	12	8	14	17	16	32	0.0001	0.0002
Flufenoxuron			1-10	55	62	71	33	42	48	88	59	48	0.0001	0.0002
Flutriafol	Linear	1/x	1-10	109	133	132	15	4	3	15	18	17	0.0001	0.0002
Imazapyr	Linear		1-10	47	22	4	30	17	14	73	77	69	0.0001	0.0002
Imazethapyr	Linear		1-10	28	43	13	128	19	11	128	64	56	0.0001	0.0002
Lufenuron	Quadratic	$1/x^2$	1-10	63	63	70	43	35	42	62	54	42	0.0001	0.0002
Methamidophos	Linear		1-10	78	66	57	6	3	6	7	3	7	0.0001	0.0002
Mevinphos			1-10	250	223	194	24	23	10	34	32	12	0.0001	0.0002
Naled	Linear	1/x	1-10	3	10	7	102	120	70	113	124	83	0.0001	0.0002
Paraoxon-methyl	Linear		1-10	98	80	96	17	8	4	21	36	16	0.0001	0.0002
Permethrin	Linear	$1/x^2$	2-20	84	93	59	69	86	87	131	86	87	0.0002	0.0004
Phorate			4-40	80	83	90	12	11	12	15	11	16	0.0004	0.0008
Picloram			4-40	15	16	2	103	89	85	145	89	85	0.0004	0.0008
Propamocarb	Linear	1/x	1-10	68	60	60	4	10	4	10	15	7	0.0001	0.0002
Propiconazole	Linear	1/x	1-10	109	99	101	46	26	7	81	44	14	0.0001	0.0002
Pyridaben	Linear	$1/x^2$	1-10	45	31	74	83	25	79	140	118	79	0.0001	0.0002
Pyrimethanil	Linear		1-10	103	136	107	21	52	42	30	60	42	0.0001	0.0002
Sulfentrazone	Linear		2-20	80	66	61	4	4	4	34	24	7	0.0002	0.0004
Temephos	Linear	$1/x^2$	2-20	70	69	73	19	30	32	45	31	33	0.0002	0.0004
Thiophanate-methyl			1-10	11	4	22	9	132	36	116	167	178	0.0001	0.0002

[1] LR: linearity range. [2] Pt: point.

Table S3. Non-approved analytes. Linearity, recovery (in %), repeatability relative standard deviation (RSD; in %), expanded measurement uncertainty (U; in %), limit of detection (LOD; in mg/kg), and limit of quantification (LOQ; in mg/kg) for each analyte of the GC-MS/MS method for analysis of pesticides in honey.

Compound	Linearity			Average Recovery		RSD		U		LOD (mg/kg)	LOQ (mg/kg)
	Type of Adjust	Ponderation	LR[1] (µg/kg)	Pt[2] 1	Pt 6	Pt 1	Pt 6	Pt 1	Pt 6		
Chlorothalonil	Quadratic	$1/x^2$	10-100	103	173	48	53	76	53	0.001	0.002
Chlorpyrifos	Linear	1/x	10-100	124	106	7	5	34	21	0.001	0.002
DDE 2,4			10-100	114	88	7	6	18	8	0.001	0.002
Dicofol			20-200	135	102	33	7	57	20	0.002	0.004
Fenitrothion	Linear	1/x	20-200	108	104	10	6	21	17	0.002	0.004
Fenvalerate	Linear	1/x	20-200	98	69	7	15	15	18	0.002	0.004
HCH beta	Linear	1/x	20-200	92	60	7	7	9	13	0.002	0.004
HCH delta	Linear	1/x	20-200	123	109	7	7	35	9	0.002	0.004
HCH gamma	Linear	1/x	20-200	64	55	32	11	93	27	0.002	0.004
Heptacloro exo epoxid	Quadratic	1/x	20-200	117	72	8	7	35	28	0.002	0.004
Metolachlor	Linear	1/x	10-100	119	101	7	4	27	16	0.001	0.002
Parathion	Linear	1/x	40-400	125	111	7	5	28	26	0.004	0.008
Parathion-methyl	Linear	$1/x^2$	40-400	126	112	7	8	36	21	0.004	0.008
Pirimiphos-methyl	Linear	1/x	10-100	130	107	8	7	35	22	0.001	0.002
Propoxur			10-100	107	94	7	6	19	9	0.001	0.002

[1] LR: linearity range. [2] Pt: point.
